# A Rare Presentation of Dual Ovarian Pathologies: Small Cell Carcinoma of the Ovary and Mucinous Ovarian Cancer

**DOI:** 10.7759/cureus.19468

**Published:** 2021-11-11

**Authors:** Syed A Mannan, Musa Azhar, Jhanzeb Iftikhar, Saad Khalil Chaudhry, Maryam Hameed

**Affiliations:** 1 Medical Oncology, Shaukat Khanum Memorial Cancer Hospital and Research Centre, Lahore, PAK; 2 Radiology, Shaukat Khanum Memorial Cancer Hospital and Research Centre, Lahore, PAK; 3 Pathology, Shaukat Khanum Memorial Cancer Hospital and Research Centre, Lahore, PAK

**Keywords:** ovarian cancer, dual primaries, gyne-oncology, mucinous ovarian cancer, small cell ovarian cancer

## Abstract

The small cell carcinoma of ovaries co-occurring with mucinous ovarian cancer is a rare event. We report a 21-years-old lady with a composite tumour comprising small cell carcinoma and mucinous carcinoma of ovaries. The incidental finding of the left ovarian cyst led to further workup and revealed a solid cystic mass in the left adnexal area pathologically proven to be mucinous ovarian carcinoma. The initial surgery was deferred upon the patient's request. After a few more cycles of chemotherapy, at the completion of surgery as per ovarian protocol, the pathological evaluation showed small cell carcinoma in the left ovary with a residual focus of mucinous carcinoma.

In contrast, the right ovary also showed surface deposits of small cell carcinoma. The patient's clinical condition deteriorated very rapidly after that, and she passed away. Early recognition of small cell carcinoma in a composite tumour is critically essential for timely intervention.

## Introduction

Ovarian carcinoma is one of the commonest gynaecological malignancies. The mucinous ovarian cancer accounts for 5%-10% of all ovarian cancers, whereas small cell ovarian carcinoma is a very rare disease with only a few hundred cases reported to date [1,2]. Small cell ovarian cancer (SCCOC) is of two types - small cell carcinoma of the ovary hypercalcemic type (SSCOHT) and small cell carcinoma of the ovary pulmonary type (SCCOPT). SSCOPT is extremely rare with only 22 cases in the literature until 2018 [3]. The SCCOC is a highly aggressive malignancy with poor survival outcomes despite aggressive treatment [1].

Here we report a unique case in which mucinous ovarian cancer and SCCOC were seen in the left ovary, whereas SCCOC was seen on the surface of the right ovary. To the best of our knowledge, there is no such case published in literature from Pakistan.

## Case presentation

**Figure 1:**
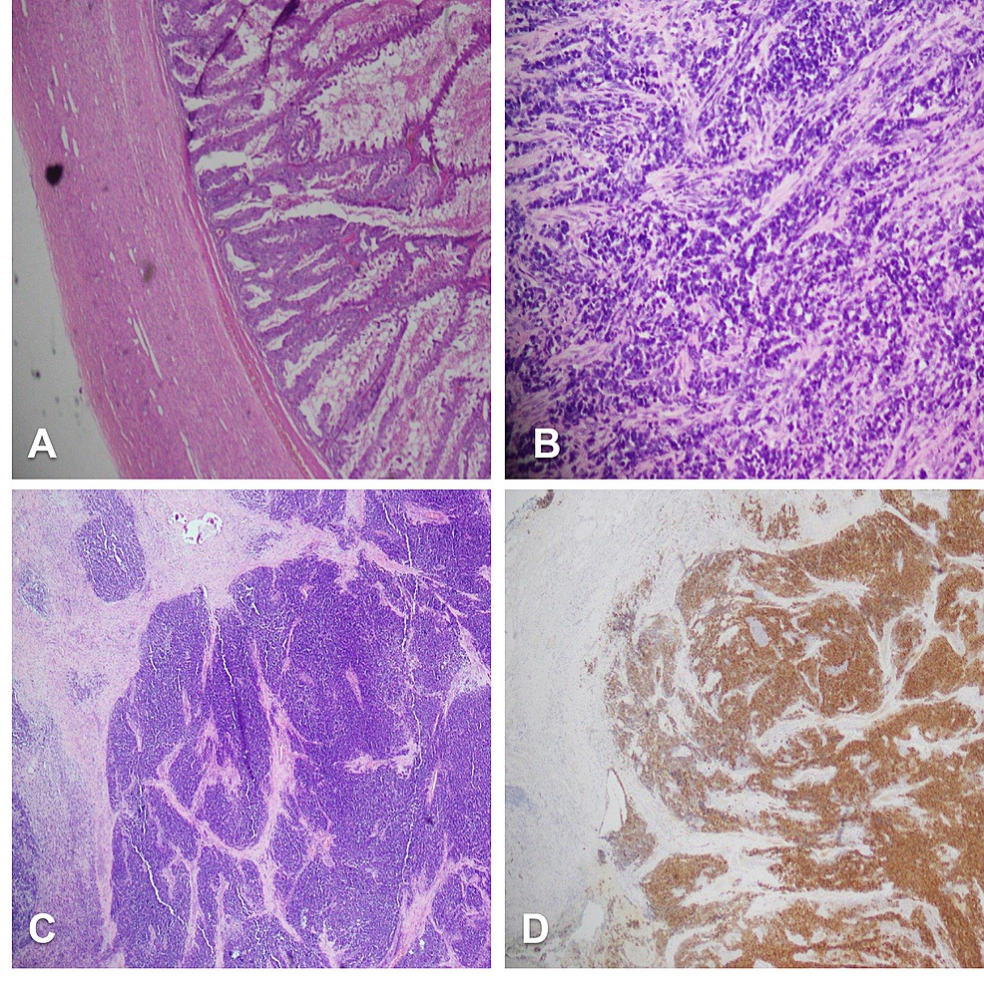
(A) Left cystectomy specimen with mucinous adenocarcinoma and high-grade cytological features. (B) Small cell carcinoma with crushed morphology and neuroendocrine-like nuclear features. (C) Left ovary in surgical specimen showing small cell carcinoma with jigsaw pattern and no significant necrotic islands. (D) Synaptophysin immunohistochemical stain highlighting the small cell carcinoma.

A 21-year-old female student of law, having a family history of breast cancer, presented to the outpatient department for further treatment after being diagnosed with stage IC well-differentiated mucinous ovarian cancer. She had a history of incidental left ovarian cyst after being worked up for abnormal facial hair. She underwent an ultrasound (USG) abdomen and pelvis, which showed a large cyst of 20 cm in size in the left ovary occupying the whole of the abdomen. Her chest, abdomen, and pelvis CT scan showed a large left complicated ovarian cyst extending up to the pancreas with the minimal obstructive ureter. Her CA-125 concentration was 21.9 U/mL. She underwent left ovarian cystectomy with laparoscopic assistance at another facility. Histopathology findings were consistent with a well-differentiated ovarian mucinous carcinoma. The immunohistochemical stains CK 7 was positive, while CK 20, CDX2, SATB2, GATA-3, GCDFP15 and mammaglobin were negative (Figure 1A).

**Figure 2:**
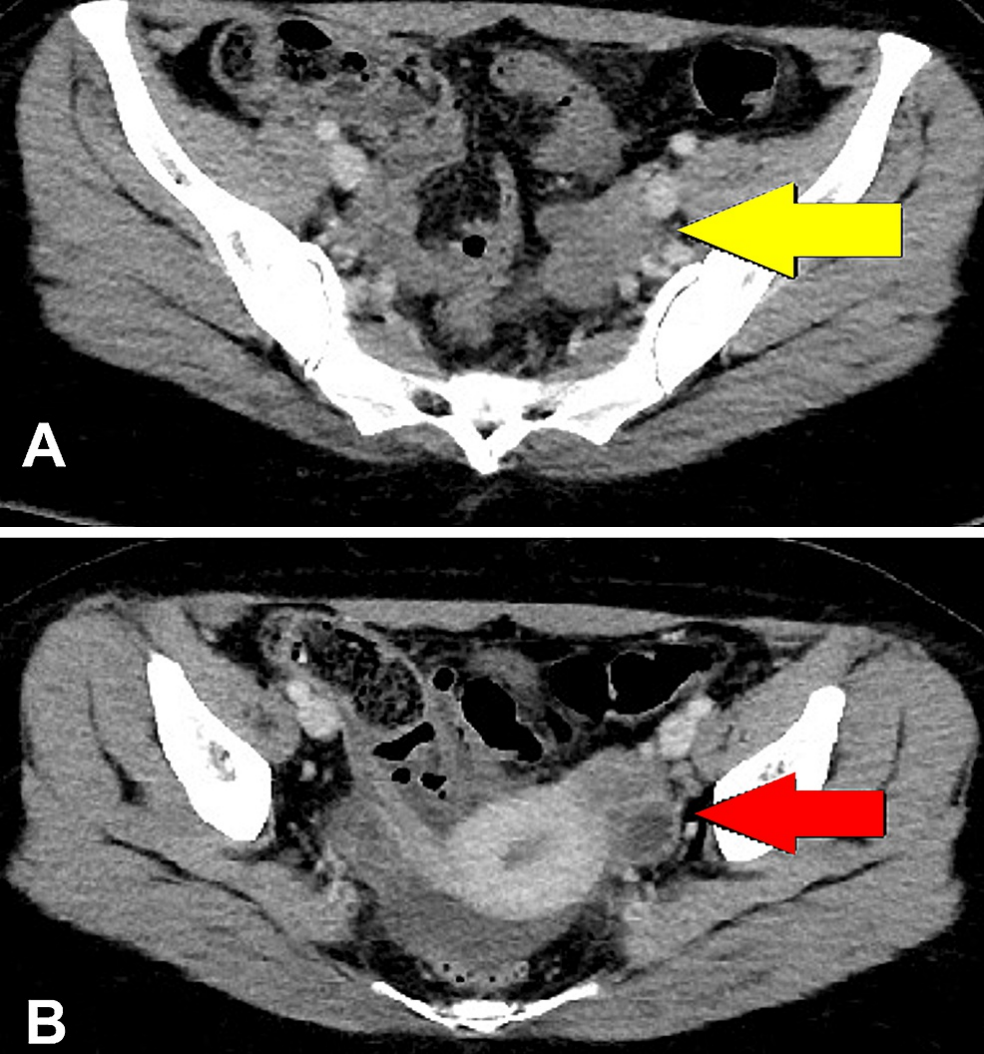
(A) Post-surgery contrast-enhanced CT scan axial image showed bulky left ovary (yellow arrow). (B) Axial contrast-enhanced CT scan through pelvis post-surgery of the same patient showed a small cyst in the left adnexa with enhancing wall (red arrow). A small amount of free fluid is also seen.

**Figure 3:**
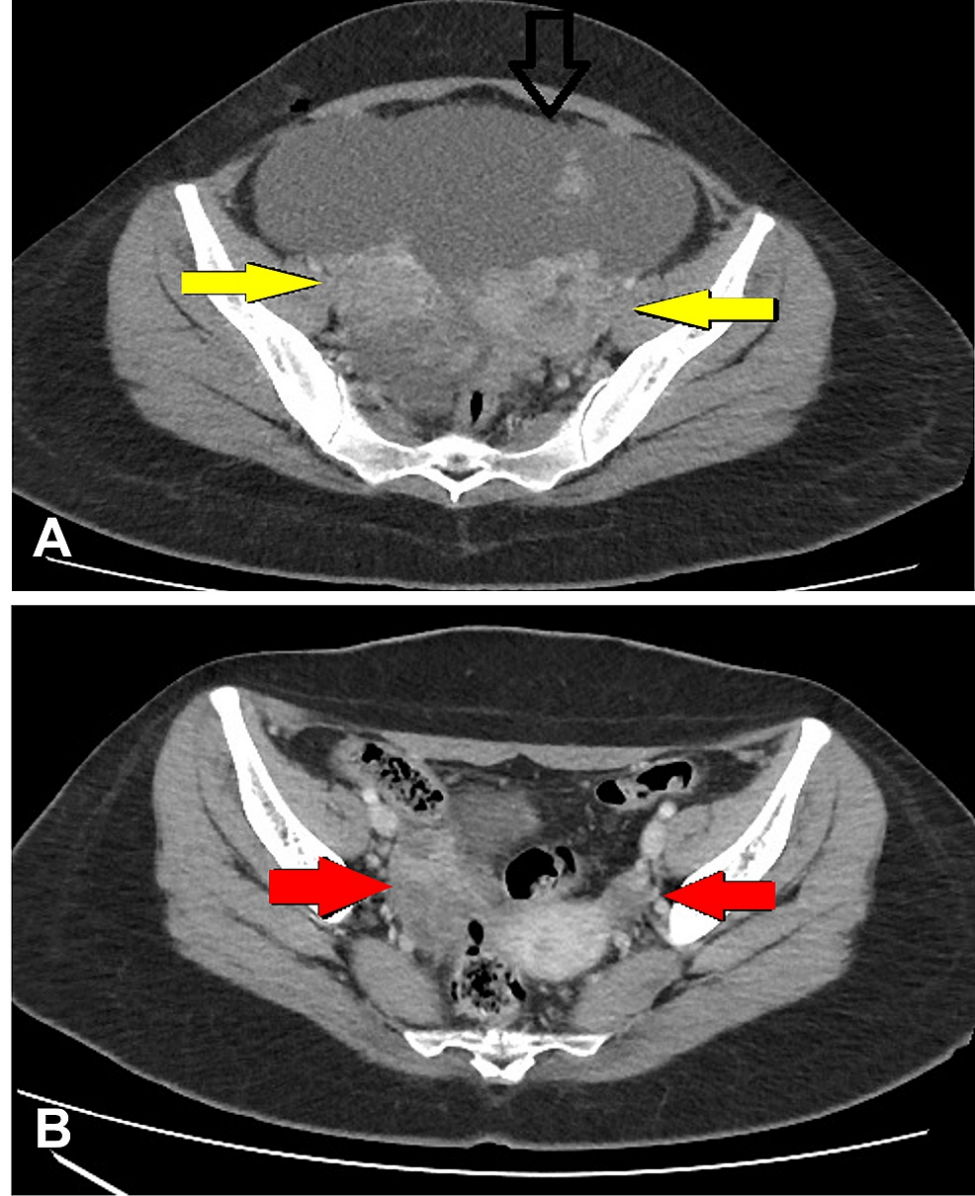
(A) Axial contrast-enhanced CT scan through the pelvis. An interim scan two months later shows disease progression with interval development of bilateral adnexal masses, ascites and peritoneal nodules (yellow arrows). (B) Axial contrast-enhanced CT scan through the pelvis. Interval treatment response status post neoadjuvant chemotherapy. There is an interval decrease in the size of adnexal masses (left more than right) and ascites (red arrows).

The ovarian capsule was ruptured during surgery; hence the FIGO stage was IC. Her post-diagnostic surgery scans are shown in Figures 2A, 2B. Her upper and lower gastrointestinal endoscopies were done, which were unremarkable. The multidisciplinary tumour board decided that she should undergo fertility-preserving completion surgery following ovarian protocol. The patient insisted on a delayed date as she had examinations. She was given a date for surgery in two months. However, she came with progressively increasing abdominal distension and few days before her surgery. Her CA-125 increased from 21.9 U/mL to 181 U/mL. The USG abdomen pelvis showed moderate to gross ascites. The peritoneal fluid cytology was positive for malignant cells. On further investigating, the CT scan of the chest, abdomen and pelvis showed disease progression with widespread omentoperitoneal disease and large volume ascites (Figure 3A). The multidisciplinary tumour board recommended deferring surgery and starting neoadjuvant chemotherapy. There was a good radiological response after five cycles of carboplatin, paclitaxel, bevacizumab, as shown in Figure 3B. She underwent completion surgery as per ovarian protocol. The histopathology report showed small cell carcinoma in the left ovary and a residual focus of mucinous ovarian carcinoma. There were surface deposits of small cell carcinoma on the right ovary.

**Figure 4:**
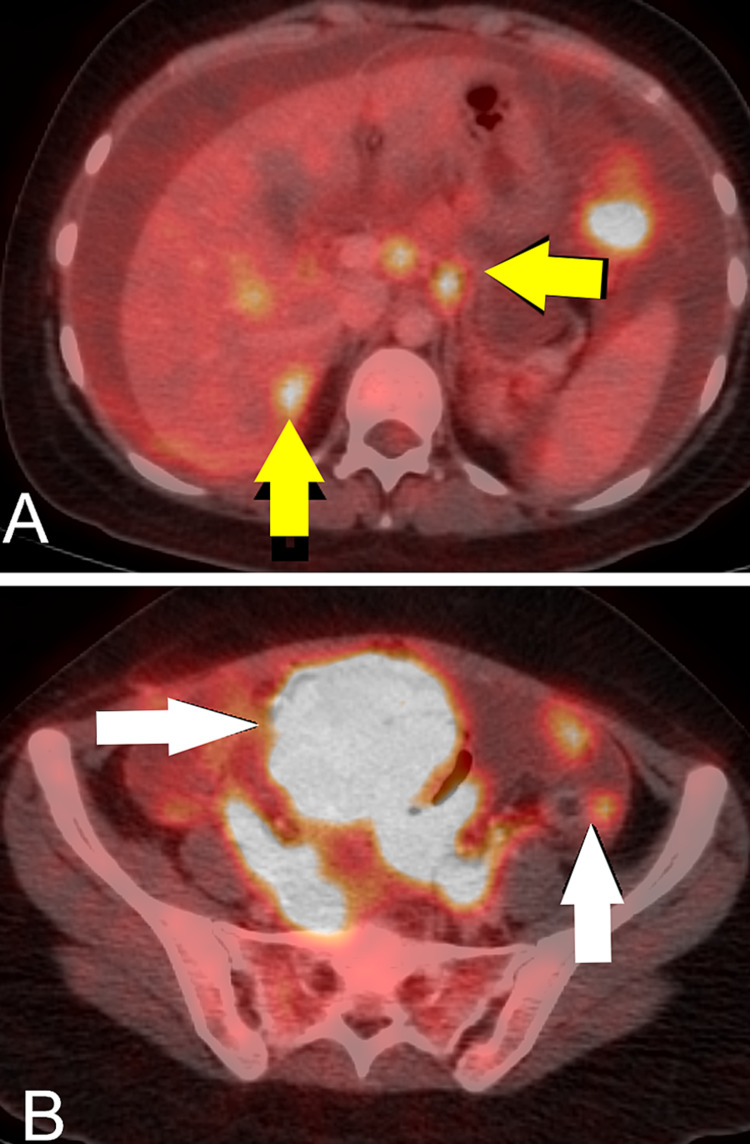
(A) PET/CT performed after surgery showed interval development of hypermetabolic hepatic lesions, portocaval nodes and ascites (yellow arrows). (B) PET/CT performed after surgery showed interval development of hypermetabolic pelvic mass with ascites and hypermetabolic peritoneal nodules along the left para-colic gutter (white arrows).

Moreover, the peritoneal wall, omentum, right fallopian tube and appendix were involved with metastatic small cell carcinoma. There was an aberrant expression of p53, and synaptophysin was positive,whileTTF1, Cam5.2, NSE, WT1, CK, LCA were negative (Figures 1B-1D). Immunohistochemistry for SMARCA4 and INI1 was performed, which were both intact. However, for confirmation of SMARCA mutation, evaluation of SMARA2 is usually required but was unfortunately not available in our institute. Considering this new pathology positron emission tomography scan was done four weeks after surgery which showed progressive disease with the development of ascites with an omental and peritoneal disease, hepatic lesion, pelvic masses and abdominal wall deposits (Figures 4A, 4B). Her serum calcium levels were 9.59 mg/dL. She was started with cisplatin and etoposide chemotherapy; however, she could receive only one cycle as her health deteriorated and she succumbed to progressive disease. From the date of her diagnosis, she could only live for 10 months.

## Discussion

Even though mucinous ovarian carcinoma is not an unusual occurrence, SCCOC with mucinous carcinoma occurs rarely. Although five such composite tumours have been documented previously, this is the first case report of synchronous tumours in either of the ovaries to our knowledge [4]. Earlier studies showed disease occurring in adult females aged 22-65 years, with abdominal distension being the predominant symptom. The tumour markers were frequently raised in previous reports. The tumour size was more than 10 cm [5,6]. In this report, the tumour was detected in a premenopausal female of 21 years, who had a history of abnormal facial hair growth and had borderline raised tumour markers.

The SCCOC has two types the pulmonary and hypercalcemic. The hypercalcemic type occurs mainly in younger females, while pulmonary type presents at an average age of 56 years [4]. The morphological features in SCCOHT and SCCOPT are usually not significantly different [7]. The hypercalcemic type may sometimes show rhabdoid morphology, comprising large cells with abundant cytoplasm and prominent nuclei [8]. On the other hand, pulmonary type shows prominent necrosis and morphology similar to small cell carcinoma of lungs [4]. SCCOHT immunohistochemistry demonstrates positivity for p53, WT1, calretinin, and CD10; SCCOPT immunohistochemistry demonstrates positivity for chromogranin, CD56, and synaptophysin [7,9]. However, there is considerable overlap in the immunohistochemistry profile of both these subtypes [2]. In our case, p53 had an aberrant expression, while synaptophysin was also positive. In 60% of SCCOHT patients, the blood calcium level is increased [10]. The calcium levels were not raised in this case. The SCCOHT is associated with a mutation in the SMARCA4 gene [1]. In almost all cases, SCCOHT displays a mutation of SMARCA4, which is a member of the SWI/SNF chromatin remodelling complex. Usually, pathologists also evaluate the loss of SMARCA2. The dual negative expression of SMARCA4 and SMARCA2 is highly sensitive and specific for SCCOHT [11]. In our case, although immunohistochemically SMARCA4 was intact, SMARCA2 was not available in our institute and therefore could not be evaluated for confirmation. Further genetic testing was not done in our case. Even though age and immunohistochemical profile favour SCCOHT, we cannot differentiate between SCCOHT and SCCOPT with confidence unless we have a genetic profile. The detection of SMARCA4 mutation confirms SCCOHT; otherwise, the possibility of SCCOPT arising in a teratoma along with adenocarcinoma cannot be entirely ruled out.

The prognosis of small cell carcinoma with mucinous ovarian carcinoma is abysmal, especially in the advanced stage. All previously reported cases died within ten months of initial presentation, even though they were not in an advanced stage at the time, and this is precisely what happened in this case. All the cases developed liver metastasis which occurred in our patient as well [4]. The treatment for SCCOC is surgery followed by chemotherapy and radiotherapy [1].

While radiotherapy is proving to be a promising treatment option in studies [1], it was not possible to use it on our patient due to her aggressive and widespread disease, which meant she did not have much time following surgery.

## Conclusions

It is infrequent but highly fatal to have both SCCOC and mucinous ovarian cancer at the same time. When it comes to managing this illness, clinical judgement and pathological examination are both critical components. Because of their great propensity to metastasize, it is crucial to identify the exact pathology as soon as possible. Any delay might be life-threatening due to their extraordinary ability to metastasize. However, the discovery of genetic flaws has opened up new possibilities in treating these cancers, even though the prognosis remains grim.
